# A critical assessment of the ideological underpinnings of current practice in global health and their historical origins

**DOI:** 10.1080/16549716.2019.1651017

**Published:** 2019-08-21

**Authors:** Hani Kim, Uros Novakovic, Carles Muntaner, Michael T. Hawkes

**Affiliations:** a Bill & Melinda Gates Foundation, Global Health, Seattle, WA, USA; b Department of Interdisciplinary Research, Office OU, Toronto, Canada; c Dalla Lana School of Public Health, University of Toronto, Toronto, Canada; d Faculty of Medicine, University of Alberta, Edmonton, Canada

**Keywords:** Global health, ideology, political origins, health equity, social determinants

## Abstract

**Background**: The current approach to global health has significantly contributed to improving it, as evidenced by the progress made toward the Millennium Development Goals (MDGs). However, the health gains achieved are often highly unequitable, and the current approach is expected to be insufficient to meet the future health equity challenges. There is an urgent need to re-think and expand the scope of research and programmatic strategies.

**Objective**: This paper aims to assess the ideological underpinnings of the currently dominant norms in global health, with the goal of highlighting the research and programmatic areas that are marginalized and warrant greater efforts in order to resolve persistent health inequity and achieve the UN Sustainable Development Goals (SDGs).

**Methods**: We have conducted a critical review of the literature that traces the historical origins of global health to the period between the mid-19th century and the end of the 20th century.

**Results**: Critical review of the historical origins of global health reveals a set of dominant norms in global health that are ideological in character, and profoundly shape the current practice. We identified key manifestations of the ideological underpinnings as 1) Democratic deficit, 2) Depoliticization of the discourse, 3) Marginalization of the scholarship that interrogates the relations of power.

**Conclusion**: Examination of the dominant norms that shape the foundation of our knowledge and action in global health is required to solve persistent health inequity challenges and meet the SDGs. Inversion of the key manifestations of the dominant norms can serve as guiding principles to elaborate alternative frameworks that have the theoretical and programmatic potential for a fundamental rather than an incremental change in the practice of global health.

## Background

### The current approach and its merits

**Figure 1.**
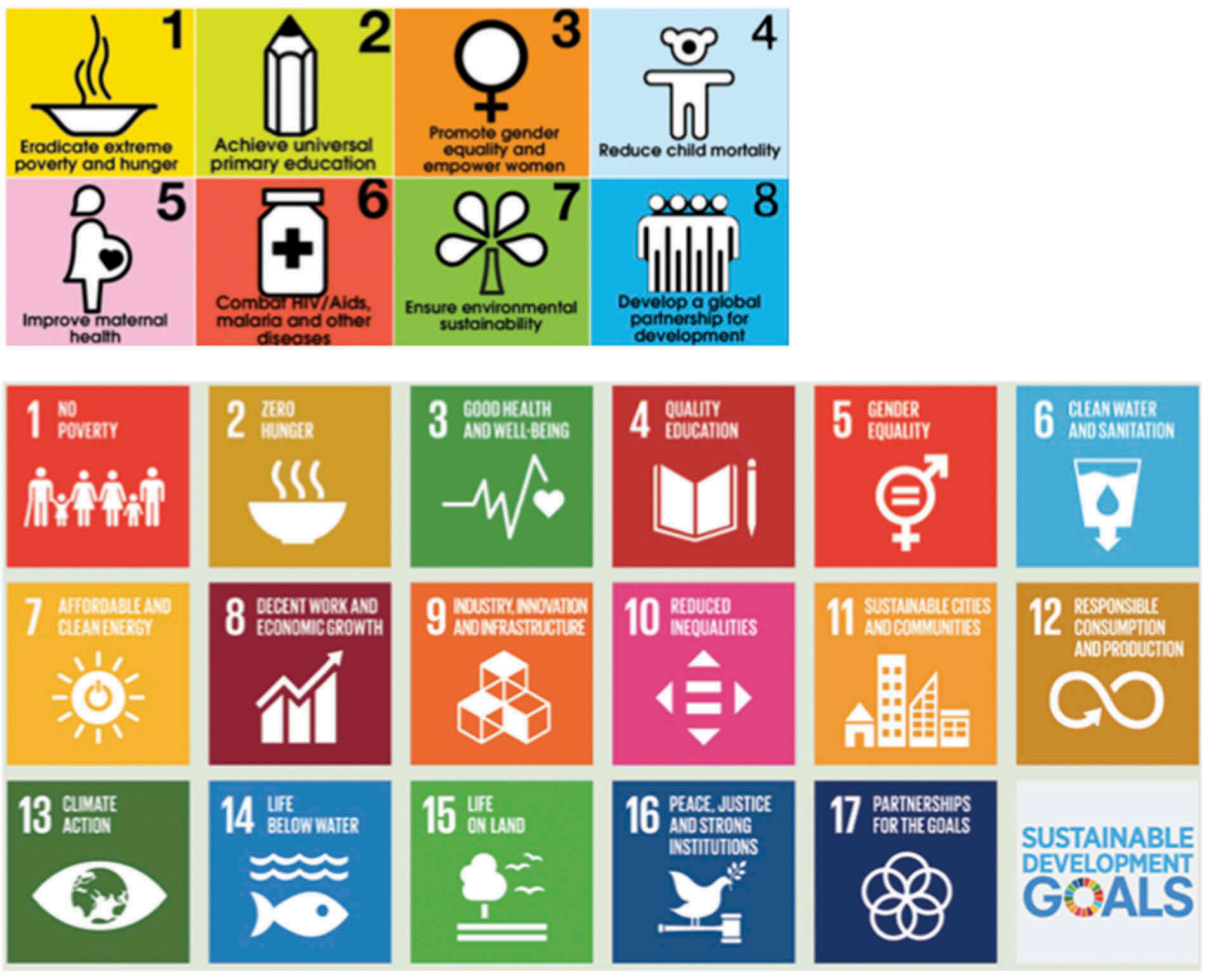
The UN Millennium development goals and sustainable development goals [10,11].

**Figure 2.**
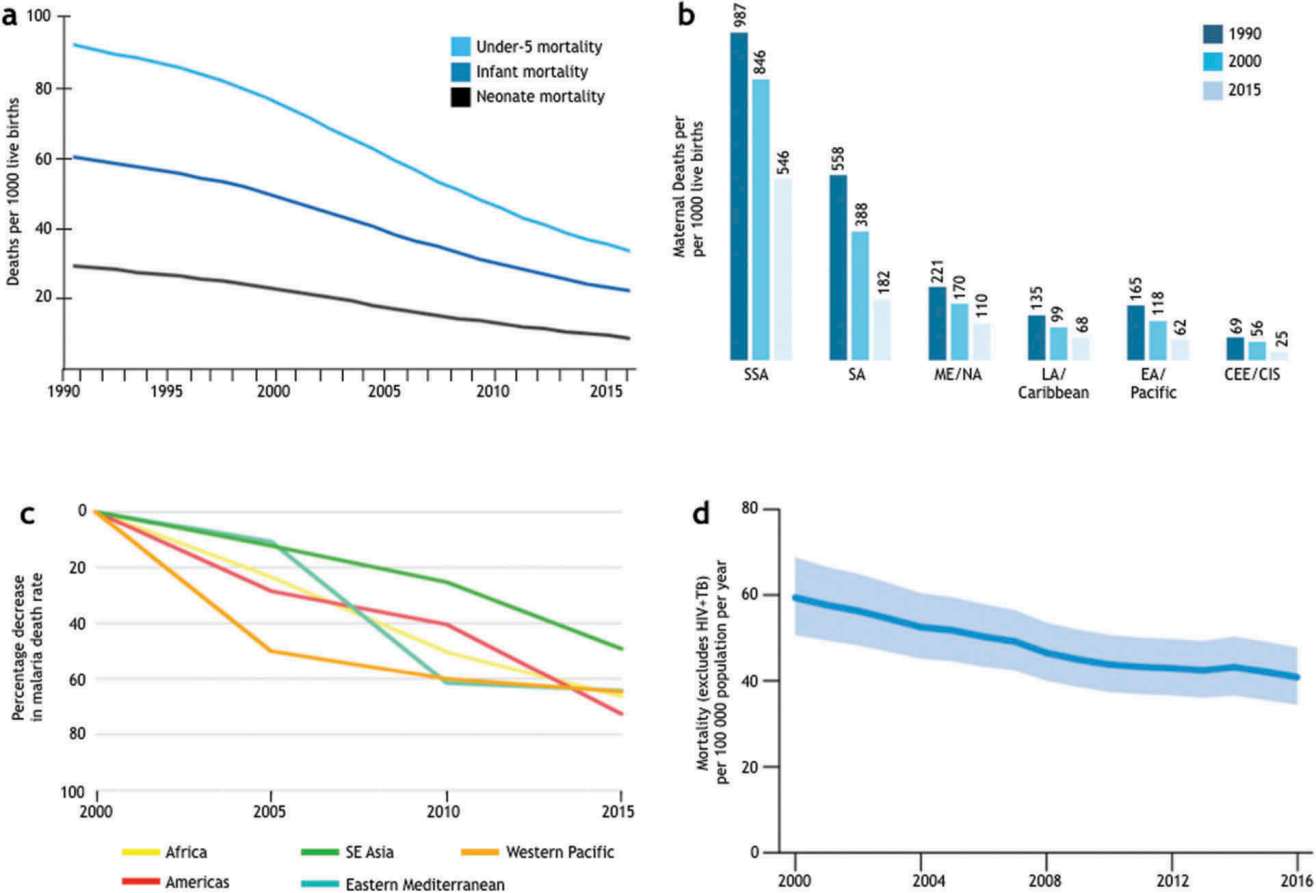
Achievements in meeting the health-related MDGs. (a) Trends in the global under-5 (top), infant (middle), and neonatal (bottom) mortality rates. Adapted from [5]. (b) Maternal deaths per 100,000 live births in women aged 15–49. CEE, Central and Eastern Europe; CIS, the Commonwealth of independent states. Adapted from [6]. (c) Percentage decrease in malaria death rate since 2000 (by WHO region) [7]. (d) Mortality associated with TB in the WHO African region between 2000 and 2016 [8].

The global health field has been grappling with defining the term ‘global health’ [1], which may reflect the multifaceted path by which the field has been evolving. For our discussion in this paper, we define it as ‘collaborative trans-national research and action for promoting health for all’, as proposed by Beaglehole and Bonita [1]. Insights into the predominant approach pursued in global health can be gleaned from the interventions used to achieve the health-related Millennium Development Goals (MDGs) between 2000 and 2015 (Figure 1). The MDGs focus on child health, maternal health and prevention and control of malaria, tuberculosis (TB) and HIV/AIDS. The declaration of the MDGs in 2000 recognized that extreme poverty is unacceptable and that eradicating it is a collective responsibility [2]. This was a normative shift in the way international development was viewed, away from a narrowly defined economic growth and toward a broader development agenda that encompasses poverty reduction, education, gender equality, and environmental sustainability [3]. Despite the broad scope of its initial aspirations, the implementation of the MDGs focused largely on targeting primarily individual-level biological causes of specific infectious diseases, such as malaria, TB and HIV/AIDS (Figure 1). Several focused interventions were identified to have contributed to achieving against the MDGs. They include immunization, insecticide treated bed-nets, antibiotics, anti-retroviral therapy, and the Directly Observed Treatment, Short-course (DOTS) for TB [4]. In addition to improved living conditions such as sanitation and access to water, these interventions have contributed significantly to improving the under-5 infant mortality, maternal mortality, and deaths due to malaria, TB and AIDS (Figure 2) [3,5–9].

The approaches reflected in the efforts to achieve the MDGs can be characterized by targeting, primarily, the *biological* causes of disease at the *individual* level to prevent (e.g. insecticide treated bed-nets to prevent exposure to mosquito bites, vaccines), and treat (e.g. antibiotics, anti-retroviral therapy) infection and/or disease. Implicit in this approach is the assumption that targeting biological causes of ill health at the individual level, especially those at an increased risk, will ultimately improve the health of a population if the individual-level interventions can be effectively scaled to a large population. This approach has contributed substantially to our ability to detect, diagnose and treat and prevent infectious diseases world-wide. The central role of medical *products* (e.g. vaccines, drugs, diagnostics) in modern medicine is emphasized in what can be termed as a product development paradigm, which can be summarized as 1) discovering, developing and delivering efficacious biomedical products that target specific individual-level etiologic agents and 2) improving managerial efficiency to optimize the process to scale a proven intervention to a large target population across different geographical locations. The contribution of these targeted interventions to the MDG health goals must not be underestimated. Nonetheless, efforts to close the gap on health equity towards the Sustainable Development Goals (SDGs) set for 2015–2030 call for critical reflection on the approaches used to achieve the MDGs (Figure 1).

### Limitations of the current approach

A growing body of evidence questions whether targeted interventions are sufficient to achieve sustainable health equity [12,13].

First, the distribution of the gains made in health outcomes during the last 19 years has proven to be far from even, with persistent inequalities or, in some cases, increased inequalities. For example, women, especially those aged 15–24, still show a disproportionately high risk of HIV infection in sub-Saharan Africa [14]. Gender norms and inequalities, insufficient access to education and sexual and reproductive health services, poverty, food insecurity and violence lie at the root of the increased HIV risk of young women and adolescent girls [14]. Childhood immunization gaps persist between and within countries, and they have been shown to be associated with household wealth, geographic location, and mother’s education [15]. In Vietnam, while the overall level of child malnutrition, measured by stunting and wasting, improved between 2000 and 2011, the concentration index revealed a significant increase in inequality in child malnutrition during the same period. Not only did the poorest quintile consistently show a greater level of malnutrition, but the difference between the poorest and richest quintiles also increased significantly during the study period [16].

Bendavid et al. recently reported decreasing inequalities in the under-5 mortality between the wealthiest and poorest tertiles in 54 low- and middle-income countries (LMICs) (e.g. Colombia, Ghana, Nepal) [17]. However, the observed convergence was highly heterogeneous, with some countries (e.g. Cambodia, Congo, Haiti, and Ukraine) showing a trend of increasing inequalities in under-5 mortality [17]. Furthermore, the report relied on demographic surveys, which cannot capture data from people who lie outside government services or surveys. An increasing number of people have been dwelling in informal settlements, the result of multiple interrelated factors, including urban-rural migration, inadequate housing, economic vulnerability, weak governance, conflicts, and wars. These people are socially and economically excluded and lie outside the reach of municipal services. Thus, the reported difference in health outcomes between the poorest and the wealthiest may be underestimated [18].

Second, lessons from the 2014–2016 Ebola outbreak in West Africa and the on-going Ebola outbreak in the Democratic Republic of Congo reveal that the targeted interventions are often disconnected from the underlying historical, political and economic causes and thus have limited, if any, impact on strengthening the health system to respond to disease outbreaks [19,20].

**Figure 3.**
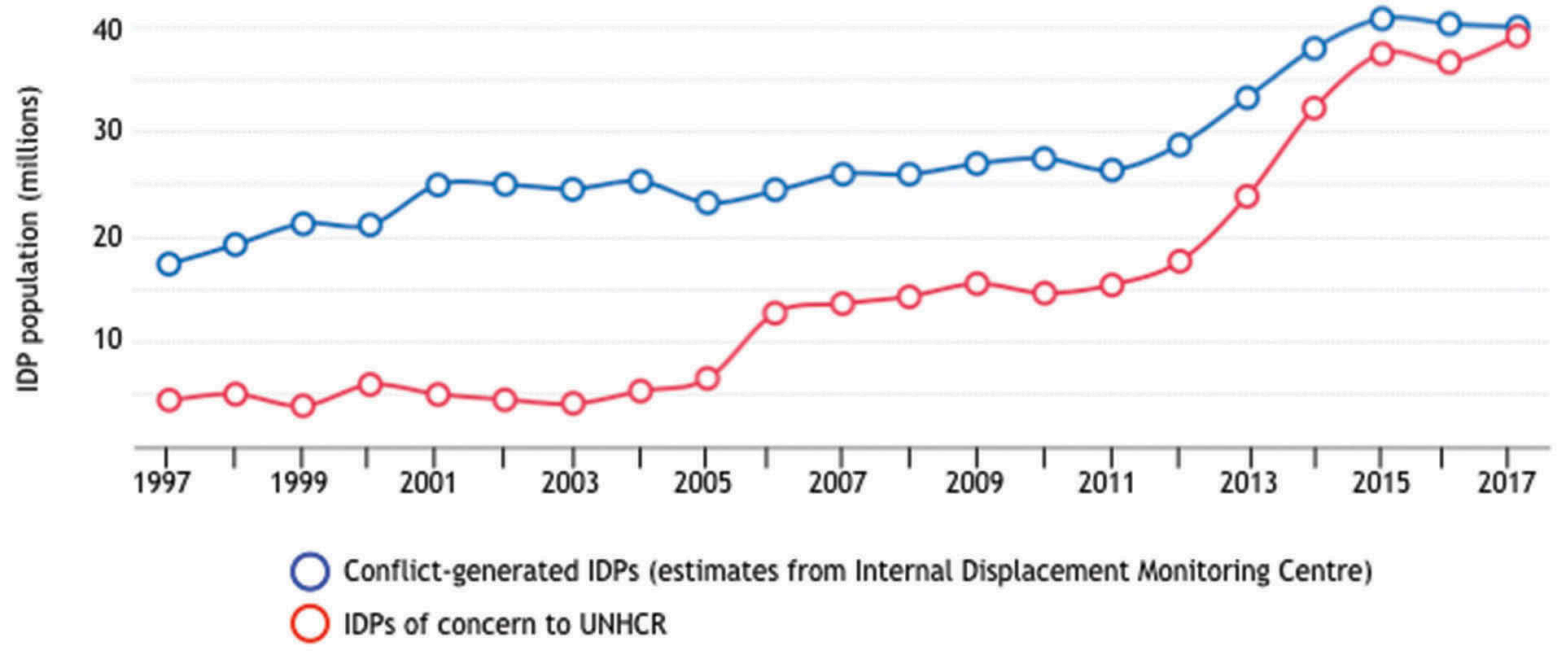
A future health inequity challenge: Forced displacement due to persecution, conflict or generalized violence [22].

Third, the current approach is expected to be insufficient to meet the looming health inequity challenges including those associated with climate change and the crisis of internally displaced persons (IDPs) due to political or economic reasons. By 2014, 60 million people had been forcibly displaced worldwide, the highest number since World War II (Figure 3) [21,22]. IDPs are structurally excluded from society and the provision of public services (e.g. health care, education, clean water). Thus, they are not only vulnerable to previously controlled diseases but also to new infections and poor mental health [23,24]. Similarly, worsening conditions from climate change are predicted to threaten the programmatic efforts for diseases that have been controlled and to increase the likelihood of new outbreaks [16,25]. Furthermore, while climate may rarely be a direct cause of conflicts, it is hypothesized that it contributes to increased conflicts and forced migration [26,27].

**Figure 4.**
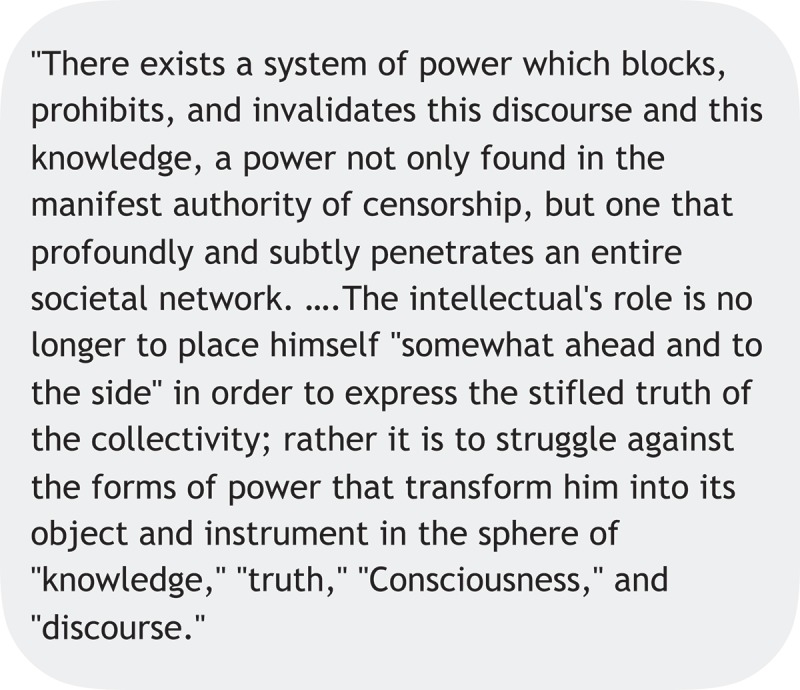
Conceptualization of ideology by Michel Foucault [29].

Independent of the merits or limitations of the current approach in meeting the future health inequity challenges, a more fundamental problem relates to the *ideological* nature of the dominant narrative governing our knowledge and practice in global health. While a large body of literature has described the history of global health, few have specifically articulated the ideological underpinnings of the current dominant norms and their manifestation. In its simplest sense, ideology is defined as ‘a system of ideas and ideals’ [28]. As conceptualized by philosophers and sociologists in the past, a system of ideas and ideals can powerfully shape what we define as ‘knowledge’, ‘truth’, or ‘discourse’ (Figure 4) [29].

Overcoming the fundamental limitations of the current approaches requires making visible the ideological underpinnings that may shape the knowledge we produce, programmatic actions we take, and the stakeholders engaged. This paper critically reviews the historical origins of global health and examines the ideological undercurrent of the dominant norms and implicit assumptions that guide our practice in global health.

## Methods

We have conducted a critical review of the literature that traces the historical origins of global health to the period between the mid-19th century and the end of the 20th century, with a hypothesis that this period has significantly shaped the ideological underpinnings of the current practice in global health.

The initial sources of data at the start of the search are the two recent, comprehensive textbooks on the history of global health, *Reimagining Global Health* (2014) and *Textbook of Global Health* (2017) [30,31]. A critical review of the two textbooks led to a refined search on specific topics to conceptualize our argument further. Search terms include, but are not limited to germ theory, cholera, Cold War, neoliberalism, Alma Ata declaration, Health for All, Selective Primary Health Care, health inequalities, health inequity, social class, and social determinants of health. The search was conducted between December 2017 and July 2019.

## Results

We report the results of our critical review of the historical origins of global health that significantly shaped the dominant norms and implicit assumptions underlying current practice based on 106 sources, as referenced.

### Birth of colonial medicine (1851–1920)

Two interrelated phenomena were critical in shaping the genesis of global health as a field in the late 1800s and the earliest approaches taken to pursue its goals: 1) the colonial programs of Europe and the USA and 2) the attendant advancement of medical science and microbiology (reviewed in [32,33]). The expansion of the reach of the 19th-century European colonial programs, as well as the rise of U.S. influence in the Americas, increased trade and commercial activities across colonial posts in Africa, Asia and South America [32–36]. The increased trading activities were accompanied by an increased transmission of infectious diseases, posing a significant threat to the colonial administration and its commercial interests [36]. For example, Cholera, which was limited to Asia until 1817, spread globally and caused six pandemics between 1817 and 1923 often following trade routes or the movements of the army troops throughout the world [37,38]. Curbing the threat of infectious diseases required the colonial nations to generate knowledge about the causes of the diseases, and to establish institutional frameworks to coordinate efforts to prevent and control disease transmission across colonial sites [34].

The desire to understand better and control the transmission of infectious diseases provided the impetus to advance our knowledge about pathogens and treatments, and gave rise to seminal discoveries of the etiologic agents and vectors of the major infectious disease of the time, including cholera (1854), malaria (1880), tuberculosis (1882), and Aedes mosquitos for Yellow Fever (1882), as well as the earliest examples of targeted treatments (e.g. quinine to treat malaria in 1897) [39–42]. The success of these scientific discoveries served as a powerful precedent to shape the way in which we were to define the ‘causes of diseases’, as expounded in the germ theory and the Koch Postulates (1890); it also paved the way to the paradigm of pathogen-specific chemotherapy seeded in Paul Ehrlich’s ‘magic bullet’(1894–1915), the notion that it is possible to identify and specifically target disease-causing microorganisms without causing harm to the host [43,44]. Contemporaneously, the earliest research institutes of tropical medicine were established, including the Liverpool School of Tropical Medicine (1893), London School of Hygiene and Tropical Medicine (1894), and the Walter Reed Army Institute of Research (1893), with their ‘field sites’ set up across sub-Saharan Africa, South East Asia and Latin America [45–47]. They remain leading institutes in global health research today.

The growing recognition of the need for international health cooperation resulted in efforts to lay out institutional frameworks that could enable cooperation among nations to harmonize and reduce the conflicting and costly maritime quarantine requirements of different colonial nations. These efforts took the form of the first international conferences, with the inaugural meeting taking place in Paris in 1851 and 11 additional conventions held between 1851 and 1903 [48]. As a result, the nascent forms of international health agencies were established, including the Pan American Sanitation Bureau (1902), a predecessor to the Pan American Health Organization; the Office International d’Hygiène Publique (OIHP, 1907); the League of Red Cross Societies (1919); and Save the Children (1920) [34].

The need to protect the commercial interests of the colonial nations in the 19th century from the increasing threat of infectious diseases provided an impetus to harness the field of microbiology and medicine, on the one hand, and to build institutions of international health governance on the other. The desire to protect the interests of *the colonizers* from *the colonized*, the infectious, placed the field of tropical medicine on a distinct trajectory from that of the public health programs of nation-states, which have a vested interest in protecting their public at large from infectious diseases. The ideas and enthusiasm espoused in the early successes in the discovery of etiologic agents and treatments during this period ‘validated’ the strategy of identifying and specifically targeting pathologic agents. The context of colonialism and the rise of modern medicine were to shape profoundly the knowledge to be generated and the programmatic strategies to be pursued, as well as the power dynamics among the stakeholders (e.g. colonizers vs. colonized, investigators vs. investigated) throughout the history of global health.

### Competing visions of international health (1946–1979)

World War II (1939*–*1945), and the subsequent context of the Cold War (1946*–*1989) represent yet another defining moment for the fledgling field of international health. The world emerged from the war deeply divided along ideological fault lines: the U.S.-led capitalist bloc (Western bloc), the Soviet-led communist bloc (Eastern bloc) and a group of non-aligned countries (also called at the time ‘the Third World’) under the Non-Aligned Movement (NAM), established in 1961 [49]. The NAM gained significant traction among the majority of LMICs at the time, with many of them emerging as nations independent from the colonial powers [50–52]. The non-aligned countries not only sought to remain independent from the influence of the USA or the Soviet Union but also proposed a distinct vision for a ‘new system of international economic relations based on equity, sovereign equality and interdependence of the interests of developed and developing countries’, as articulated in the New International Economic Order [53].

The Cold War ideologies espoused by the three blocs exerted a powerful influence in shaping the strategies and policies of post-war reconstruction, as well as international development efforts, including health, with the USA and the Soviet Union often competing to gain influence over the non-aligned countries [51]. The ideological divide manifested itself in competing visions to achieve health and health equity: 1) the market-delivered health services championed by the USA, 2) the centralized, state-led provision of primary health care (PHC) championed by the Soviet Union, and 3) the community-driven, government-led ‘health for all’ approach aspired mainly by the LMICs. These competing visions were most evident in two historical moments during the Cold War: the small pox eradication campaign and the Alma Ata Declaration to achieve Health for All by 2020.

Nearly a decade after the failure of the global malaria eradication campaign of 1955–1969, the World Health Organization (WHO) declared the success of the smallpox eradication campaign with the last case of smallpox detected in 1978 [54]. The two global disease eradication campaigns drew critical reflections on disease eradication campaigns within the field, with concerns about the significant financial burden placed on the governments of LMICs and their tendency to divert the health expenditure of local governments away from other basic health infrastructure (e.g. water, sanitation, safe housing, education) [52,55,56]. Nonetheless, the contested success of smallpox eradication at the time was later to be heralded as the success of a technologically-based, disease-focused intervention [33,57–59].

In the same year of the smallpox eradication (1978), aspirations to achieve ‘health for all by 2020’ culminated in the signing of the Alma Ata Declaration by 134 countries at the International Health Conference on Primary Health Care, convened by the WHO and UNICEF and hosted by the Soviet Union in Alma Ata, Kazakhstan (then part of the Soviet Union) [60]. The Alma Ata Declaration defines PHC as ‘essential health care based on practical, scientifically sound and socially acceptable methods and technology made accessible to individuals and families’ as part of ‘a comprehensive national health system’ [60]. The Alma Ata Declaration reflects the vision of the NAM, and it was distinct from the centralist, state-led PHC advocated by the Soviet Union or the market-delivered PHC advocated by the USA. Instead, it emphasizes ‘maximum *community* and *individual* self-reliance and participation’ while acknowledging governments’ responsibility for the provision of adequate health care [60]. It further emphasizes delivery of Health For All in alignment with the ‘economic and social development based on the New International Economic Order’ [53,60].

The Alma Ata Declaration is perceived as a victory in international health for the LMICs in its quest for a transformation in the power structures to achieve health equity [61–63]. On the other hand, critics argued that it had failed to articulate how the PHC scale-up would be financed and implemented worldwide, and that ‘its very scope makes it unattainable because of the cost and numbers of trained personnel required’ [64]. However, in the face of the billions of dollars of military spending by high-income countries during this period, as noted by Ted Kennedy, a U.S. senator at the time [65], one could question whether it was the lack of financing plans or lack of political will that undermined the efforts to materialize the vision of the Alma-Ata Declaration.

Between 1946 and1979, different visions to achieve health were articulated, each with its legitimacy supported by its own success [34]. The global context after World War II meant that the differences between the visions were deeply ideological in nature, and thus, were arguably irreconcilable from the start. The subsequent trajectories of the competing visions were largely to be determined by the battle for hegemony between the USA and the Soviet Union.

### The rise of neoliberalism (1979–2000)

By virtue of their association with the configuration of power during the Cold War, the competing visions to achieve health and their relative power to influence the direction and strategies of international health were not on an equal plane: the community-oriented PHC movement, largely promulgated by the non-aligned countries, was sidelined in the battle for hegemony between the Soviet Union and the USA. In the years following the Alma Ata Declaration, two closely related political currents were to turn the tide decisively against the vision of Alma Ata: the collapse of the Soviet Union and the Eastern bloc (1989–1991), and the ascendancy of a set of economic and political ideas known as neoliberalism.

Dating back to at least 1946, advocated chiefly by two Nobel laureate economists, Friedrich von Hayek and Milton Freedman, neoliberal values emphasize individual freedom, with the freedom of the market and the entrepreneurial spirit of individuals as central to ensuring it [66]. Human welfare can thus, be best advanced by ‘liberating individual entrepreneurial freedoms and skills by strong private property rights, free markets and free trade’ [66]. It follows, the social good, including health, would be best achieved by maximizing the reach and efficiency of market transactions. The collapse of the Eastern bloc signified the triumph of the West’s liberal democracy and the values embedded in it, to which the ideal of individual liberty is deeply tied.

Aside from the shifting configuration of power, with the USA emerging as the world’s biggest superpower, the economic recession for the industrialized countries in the 1970s (including the USA and the UK) further legitimated the neoliberal values [66,67]. Characterized by stagnation in economic growth and high level of inflation and unemployment, this period of ‘stagflation’ challenged the principles of Keynesianism which advocate for government interventions to stabilize the economy against the boom-bust cycle by actively regulating fiscal and monetary policies and opened the way for neoliberal theories to come to the fore as the main alternative. By 1978, Deng Xiaoping took the first formal steps to liberalizing China’s economy; Margaret Thatcher and Ronald Reagan, elected in 1979 and 1980 respectively, brought in broad sets of policies to deregulate and privatize the economy and reduce the role of the state in the areas of social provision.

The most striking way in which neoliberal policies directly affected health in the LMICs has been through the structural adjustment programs (SAPs) prescribed by the international financial institutes (IFIs): the International Monetary Fund (IMF) and the World Bank. SAPs were developed by IFIs as a tool to help governments restructure their economies to control inflation, repay international debt and stimulate economic growth [68]. The IFIs provide loans and debt relief to target countries under a set of specific conditions that demand 1) the promotion of the free markets, 2) the privatization of state industries, 3) economic deregulation, and 4) small government [69]. By 1991, 75 of the poorest countries in the world received adjustment loans, 30 in Africa and at least 18 in Latin America [70]. Conclusive findings on specific population-level health outcomes have been controversial due to the inherent challenge of disentangling multiple factors in a nation’s health system. Nevertheless, a body of literature has compellingly argued for the negative impact of structural adjustments on income and social inequalities and on health indicators throughout Africa in the 1990s (Reviewed in [68,71–75]). It is hypothesized that the adjustment policies harm public health by cuts to public sector services, such as health care, education, agriculture, water and public works, and the imposition of fees for health care services [69]. Growing discontent with the SAPs and with rising economic inequalities in the 1980s and the 1990s was among the key drivers for the declaration of the MDGs in 2000 [3].

Chronic funding scarcity in international health, exacerbated by the economic recession in the 1970s and the 1980s, led to a concern about financing international development and health. In 1979, as an alternative to PHC, two researchers at the Rockefeller Foundation presented ‘Selective Primary Health Care: An Interim Strategy for Disease Control in Developing Countries’ at a conference held in Bellagio, Switzerland [64,76]. The role of the Rockefeller Foundation in unveiling Selective Primary Health Care (SPHC) is consistent with its status as the single most influential funder in global health throughout the early 20^th^ century [77]. Briefly, the SPHC consisted of four interventions known as GOBI: 1) Growth monitoring, 2) Oral rehydration, 3) Breast-feeding, and 4) Immunization [61,64]. The main rationale for SHPC was the ease of monitoring and evaluating the results and clear targets [61,64]. The report initially caused a heated debate, polarized between comprehensive or ‘horizontal’ health care vs. selective or ‘vertical’ care [76]. However, by 1984, this debate was settled in favor of the cost-effectiveness model embodied by the SPHC. With strong champions in the U.S. government and the World Bank (with a former Secretary of the State of the U.S. government as its president), UNICEF spearheaded the implementation of the SPHC between 1984 and 1990 [61,78]. Arguably, the appeal of the cost-effectiveness model to donors was further enhanced by the looming threat of the new epidemic of HIV/AIDS, with detection of the first case of AIDS in 1981 [79,80].

The shifted configuration of power at the end of the Cold War, the rise of U.S. hegemony and neoliberal values combined with chronic funding scarcity and the threat of the HIV/AIDS to turn the tide decisively against the vision of Health for All reflected in the Alma Ata Declaration and in favor of a set of narrowly focused cost-effective interventions.

## Discussion

### Ideological underpinnings of current practice of global health

A critical review of the historical origins of global health reveals the shaping of a set of dominant norms and implicit assumptions that undergird current practice in global health. We posit that the dominant narrative displays three key manifestations that are ideological in character:1) a democratic deficit in the decision-making processes of research and programmatic actions, 2) the depoliticization of the discourse in health equity, and 3) the marginalization of scholarship that interrogates relations of power as a determinant of health equity. These manifestations serve as powerful currents to perpetuate the dominant norms and restrict the scope of our thoughts, strategies, and actions. We closely examine the ideological underpinnings of these manifestations below.

#### Democratic deficit

Arguably, the most far-reaching manifestation of the dominant norms in the global health field is the democratic deficit in the processes of making decisions on the scope of research and programmatic activities [12]. This profoundly influences the production of knowledge and practice in the field. Under the neoliberal framework, the desire to maximize individual liberty and rights is at odds with governance by majority rule [81]. Thus, a neoliberal approach favors governance by experts and elites, whose power is protected in insulated institutions. In global health, the anti-democratic tendencies of neoliberalism meet 19th-century colonial legacies, which inherently privilege the colonizers over the colonized.

Significant power disparities exist *among* and *within* different stakeholder groups, such as governments, researchers, civil society organizations, private philanthropies (e.g. the Bill and Melinda Gates Foundation) and multinational pharmaceutical companies [82]. An asymmetry in decision-making power is observed not only *between* stakeholder groups (e.g. governments vs. NGOs, public institutions vs. multinational corporations) but also *within* each stakeholder group (e.g. academic researchers in high income countries vs. those in LMICs, political and technical elites vs. the working-class communities within LMICs). These stakeholders hold vastly different levels of power and thus different degrees of control and ownership of decision-making processes, ranging from determining research questions, programmatic priorities, relative amount of resources to be allocated, and the stakeholders to be engaged. Indeed, a recent article reported that the vast majority of PubMed titles with ‘global health’ are from northern institutions [83], which raises the question of whether global health is a concept largely driven and owned by the high-income countries in the northern hemisphere. The dominant norms governing the practice of global health tend to concentrate the control of the decision-making processes within a group of technical experts and elites. The democratic deficit in global health governance has stimulated an animated discussion [12,77,82,84,85] that points to the importance of ‘bottom up (health) activism’ to deepening the democratic character of global health governance [84,85].

The deleterious impact of the SAP on health illustrates how neoliberal policies can significantly erode democracy in the practice of global health. In their pursuit of privatizing public services and weakening governments and public services, the SAPs redirected foreign aid from major donors away from the local governments of LMICs to Nongovernmental Organizations (NGOs) [68]. Because donors often bypass national and local health authorities in favor of international NGOs, they exert undue influence in shaping national health programs and distort the health priorities in such a way that they may not accurately reflect the interests and the wishes of the local population [19,20,77,86]. By one conservative estimate, only 11.5% of resources appropriated for the emergency Ebola response were channeled through the governments of the three most affected countries [19,87]. While the frequently cited concerns with corruption and mismanagement are not invalid, bypassing the local public health authorities in favor of international NGOs contributes to a weakening of the local public health system. Concerted efforts to collaborate with and support the local governments are critical, even in times of emergency outbreak response [19].

#### Depoliticization of the discourse

The dominant norms observed in the practice of global health tend to drive the discourse and action toward a methodological individualism that focuses on individual-level biological causes, attributes, or risk factors, which lend themselves as ‘targets of intervention’. This view is associated with search for behavioral or technological interventions, which are detached from the broader context of political economy [88].

The depoliticization of the discourse in global health is also manifested in a disproportionate emphasis on technological solutions. The roots of this fetish for commodifiable, product-driven solutions can be found in the neoliberal framework, in which ‘products of innovation’ (i.e. new products, new production methods, and new organizational forms) are a desirable consequence of individual entrepreneurial freedom [81]. Within the neoliberal framework, the proliferation of products of innovation is deeply tied to its own definition of success in materializing human welfare. When this notion becomes implicitly or explicitly dominant, there is a danger of assuming that there is a technological fix for everything and of discrediting a theoretical approach if there is no easy technological fix. Encouragingly, a recent report from the UK suggests that the biomedical R&D industry may have reached the point of diminishing return. The report calls for a ‘radical shift of life sciences funding priorities from the biomedical bubble and towards the social, behavioural, environmental determinants of health’, and for a vision of public engagement that can influence high-level strategies [89,90].

While much of the literature on the social determinants of health treat individual-attributes or risk factors as a way to contest the assumed primacy of technological, product-driven solutions to health problems, this is a false dichotomy. The risk-factor epidemiology assumes that it is individuals’ responsibility, and therefore simply a matter of exercising their discipline and control over their behaviours to avoid known risk factors regardless of a broader context within which individuals must exercise their agency [43,91–93]. This view is consistent with the neoliberal prioritization of individual liberty and responsibility over the collective responsibility. Whether the focus is on biological causes or individual-level ‘social, behavioural determinants’, both approaches represent methodological individualism, which treats individual attributes as isolated, apolitical variables that are independent of the surrounding social and political processes, thus contributing to depoliticizing the discourse.

#### Marginalization of the scholarship that interrogates the relations of power

Depoliticizing health equity problems precludes, consciously or subconsciously, an examination of the political and economic contexts within which individuals exercise their agency. As such, it prioritizes approaches that generate commodifiable intervention products and discredits theoretical constructs that are not easily manipulatable in the current policy space [94–96]. For example, two individuals of the same sex, the same level of income and the same level of education can face vastly different strategies and choices – i.e. different scopes within which they can exert their agency, to survive and flourish – in contexts that differ in employment condition; access to welfare programs; exposure to stigmatization, exploitation, or domination; and degree of control or ownership of all productive resources relevant to physical and mental wellbeing. In other words, the depoliticized, individual attributes (e.g. education, sex, age, race) possess limited explanatory power for the social *mechanisms* that generate health inequalities and offer little guidance for social policies [97]. In addition to the individual attributes, concepts such as social class, exploitation, domination, control and ownership of resources and decision-making power are valuable constructs through which one can generate insights about the social mechanisms underlying health inequalities. However, these concepts, and scholarship that interrogates relations of power as a determinant of health inequity, are marginalized in the current discourse of global health [88,98–103]. Stifling the discourse that interrogates the relations of power serves to legitimate and perpetuate the dominant norms and their associated manifestations described above. As a notable example, while the report of the WHO Commission on Social Determinants of Health [104] emphasizes the problem of unequal distribution of power as a critical determinant of health, it explicitly avoids the issue of social class and is limited to individual attributes. As a result, the report offers few insights into economic and power inequalities and how they may give rise to health inequity [99,105].

### A way forward

Overcoming the persistent health equity challenges and achieving the SDGs require a radical shift in the way we view and generate hypotheses about the mechanisms by which health inequity is generated in specific contexts. What is needed then is a set of principles that could guide us on the path of re-examining the dominants norms governing our practice in a fundamental way rather than seeking incremental modifications to the current programs and policies.

**Figure 5.**
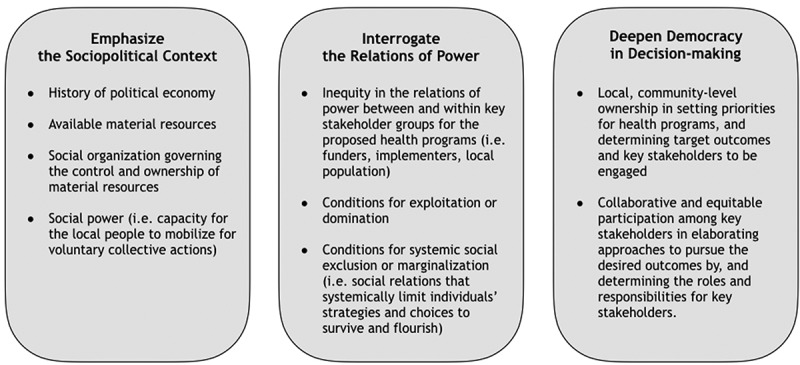
Proposed principles to guide designing of health programs.

We have identified a set of key manifestations of the dominant norms that undergird current practice in global health. We propose the inversion of three manifestations of these norms to serve as a compass, i.e. a set of guiding principles to design or evaluate health programs in specific contexts (Figure 5).

*Situating the problem in the full social and political context* requires analyzing the context of the social and political processes within which a proposed program is to be implemented. Determining the target outcomes and the hypothesized mechanisms by which the target outcomes are to be achieved should be elaborated in this context.

*Interrogating the relations of power* implies the importance of an active inquiry into the relations of power among not just the immediate stakeholders of the proposed program but also the local population at large, which the study population is assumed to represent. Such an inquiry can reveal hidden social processes that may underlie health and health equity problems observed in the chosen population, including social exclusion of particular social group(s), domination, and exploitation.

*Deepening democracy in decision-making* emphasizes the need to share decision-making power among the key stakeholders when setting priorities for health programs and determining the target outcomes for the proposed programs, desired approaches and the hypothesized mechanisms by which the proposed approaches should achieve the target outcomes.

The proposed principles are intended to serve as a *compass*, not a blueprint for all health programs in all contexts. It does not prescribe or preclude specific requirements, technical or non-technical, for a program to achieve its target outcomes (e.g. high-quality data on vaccine effectiveness to guide national policy on routine immunization programs). Instead, it calls into question the foundational assumptions and norms that may hinder our view as we imagine overarching programmatic strategies and scope. It will thus help us to elaborate on proposed health programs’ specific contexts in a manner that is cognizant of the conscious and subconscious undercurrents that tend to keep us in an intellectual and programmatic ‘business-as-usual’ mode.

## Conclusion

A radical shift in thinking about programmatic strategies in global health is possible only when we critically examine the set of norms and assumptions that shape the very foundation of what we produce as ‘knowledge’ and the programmatic actions that are developed from such knowledge. Tracing the historical origins of the global health field illuminates the arc along which the currently dominant norms in the field have been shaped since the birth of colonial medicine since the mid-19th century.

We posit that the manifestations of the dominant norms identified are ideological in character and profoundly influence (consciously and subconsciously) the programmatic strategies and scope in global health. Inverting these manifestations could help us to elaborate alternative approaches and theoretical frameworks that might help us to re-orient ourselves in a fundamental rather than an incremental way.

## Paper context

Although previous literature has described the history of global health, narrating historical events is distinct from dissecting the ideological undercurrents that emerge from them. To our knowledge, few have specifically articulated the relationship between the historical origins of global health and a set of dominant norms and assumptions that lie at the foundation of current practice. We identified the key manifestations of the dominant narrative and propose guiding principles for designing health programs.
